# First detection and prevalence of *Apis mellifera* filamentous virus in *Apis mellifera* and *Varroa destructor* in the Republic of Korea

**DOI:** 10.1038/s41598-024-64882-z

**Published:** 2024-06-19

**Authors:** Thi-Thu Nguyen, Mi-Sun Yoo, Hyang-Sim Lee, A-Tai Truong, So-Youn Youn, Se-Ji Lee, Jaemyung Kim, Yun Sang Cho

**Affiliations:** 1 Laboratory of Parasitic and Honeybee Diseases, Bacterial Disease Division, Department of Animal and Plant Health Research, Animal and Plant Quarantine Agency, Gimcheon, 39660 Republic of Korea; 2 Institute of Biotechnology, Vietnam Academy of Science & Technology, Ha Noi, 11300 Viet Nam; 3 Faculty of Biotechnology, Thai Nguyen University of Sciences, Thai Nguyen, 250000 Viet Nam

**Keywords:** Pathogens, Entomology, Viral pathogenesis

## Abstract

*Apis mellifera* filamentous virus (AmFV) is a double-stranded DNA virus that infects *Apis mellifera* bees. To our knowledge, this is the first comprehensive study aiming to detect and analyse the genetic diversity and prevalence of AmFV in Korean honeybee colonies. Phylogenetic analysis based on baculovirus repeat open reading frame–N gene (*Bro*–N) sequences revealed that AmFV isolates from the Republic of Korea (ROK) fell into two distinct lineages, with genetic origins in Switzerland and China, with nucleotide similarities of 98.3% and 98.2%, respectively. Our prevalence analysis demonstrated a noteworthy infection rate of AmFV in 545 honeybee colonies, reaching 33.09% in 2022 and increasing to 44.90% by 2023. Intriguingly, we also detected AmFV in *Varroa destructor* mites, highlighting their potential role as vectors and carriers of AmFV. The presence of AmFV was correlated with an increased infection rate of sacbrood virus, deformed wing virus, Lake Sinai virus 2, black queen cell virus, and *Nosema ceranae* in honeybee colonies. These findings provide valuable insight into the prevalence and potential transmission mechanisms of AmFV in honeybee colonies in the ROK. The results of this study may be instrumental in the effective management of viral infections in honeybee apiaries.

## Introduction

Honeybees (*Apis mellifera*) play a crucial role in pollination and sustainable maintenance of global ecosystems. Hence, their health and survival are vital for agriculture and biodiversity^1^. However, honeybee populations worldwide face numerous threats, including the emergence of viral pathogens that contribute to colony loss^2–6^. *Apis mellifera* filamentous virus (AmFV), which belongs to the family Flaviviridae, causes a viral disease that adversely affects honeybee health^5–11^. The viral particle is a double-stranded DNA molecule encapsulated as a single filament coiled within a membrane^12,13^.

AmFV was first identified in honeybees in the United States (US) and was subsequently reported in various countries across different continents, including Switzerland, France, Sweden, China, Syria, the Czech Republic, Mexico, Argentina, and Hungary^8–11,14–19^. AmFV infection can lead to a reduced honeybee lifespan, impaired brood development, and compromised colony health. It is detectable year-round, with higher viral copy numbers typically observed in spring^7^. AmFV has been detected in both honeybees and solitary bee species^19,20^. Understanding the prevalence and genetic diversity of AmFV is crucial for implementing effective management strategies, to mitigate its impact on honeybee populations.

AmFV infection in honeybee colonies produces various symptoms, including milky-white hemolymph in severely infected adult bees, dead bees, and crawling bees at hive entrances, and often results in colony loss^9,12,14^. AmFV has been found to have a greater potential for infecting adult honeybees when they are already infected with *Nosema apis*^10^. The relationship between AmFV and black queen cell virus (BQCV) has also been reported^10,21^. Additionally, Hartmann et al. reported that indicated a high positive correlation between AmFV loads and Deformed wing virus (DWV-A), Sacbrood virus (SBV), and BQCV^7^. The presence of various diseases, including viruses, parasites, fungi, bacteria, and protozoa^2–5^, particularly species like *Nosema ceranae* and *N. apis*^10,22,23^, are considered to contribute factors of colony collapse disorder (CCD) in honeybees. Although no direct link between AmFV and CCD has been established, the high prevalence rate and its potential relationship with honeybee pathogens raise concerns about the role of AmFV as a potential pathogen in honeybees. Understanding the distribution and prevalence of AmFV in honeybee populations becomes crucial for developing effective management strategies and controlling its spread. This includes the development of diagnostic tools to detect the virus and investigation of potential treatments and control measures. The use of polymerase chain reaction (PCR) has facilitated the detection of AmFV in honeybee populations worldwide, leading to an improved understanding of its distribution and prevalence.

**Figure 1:**
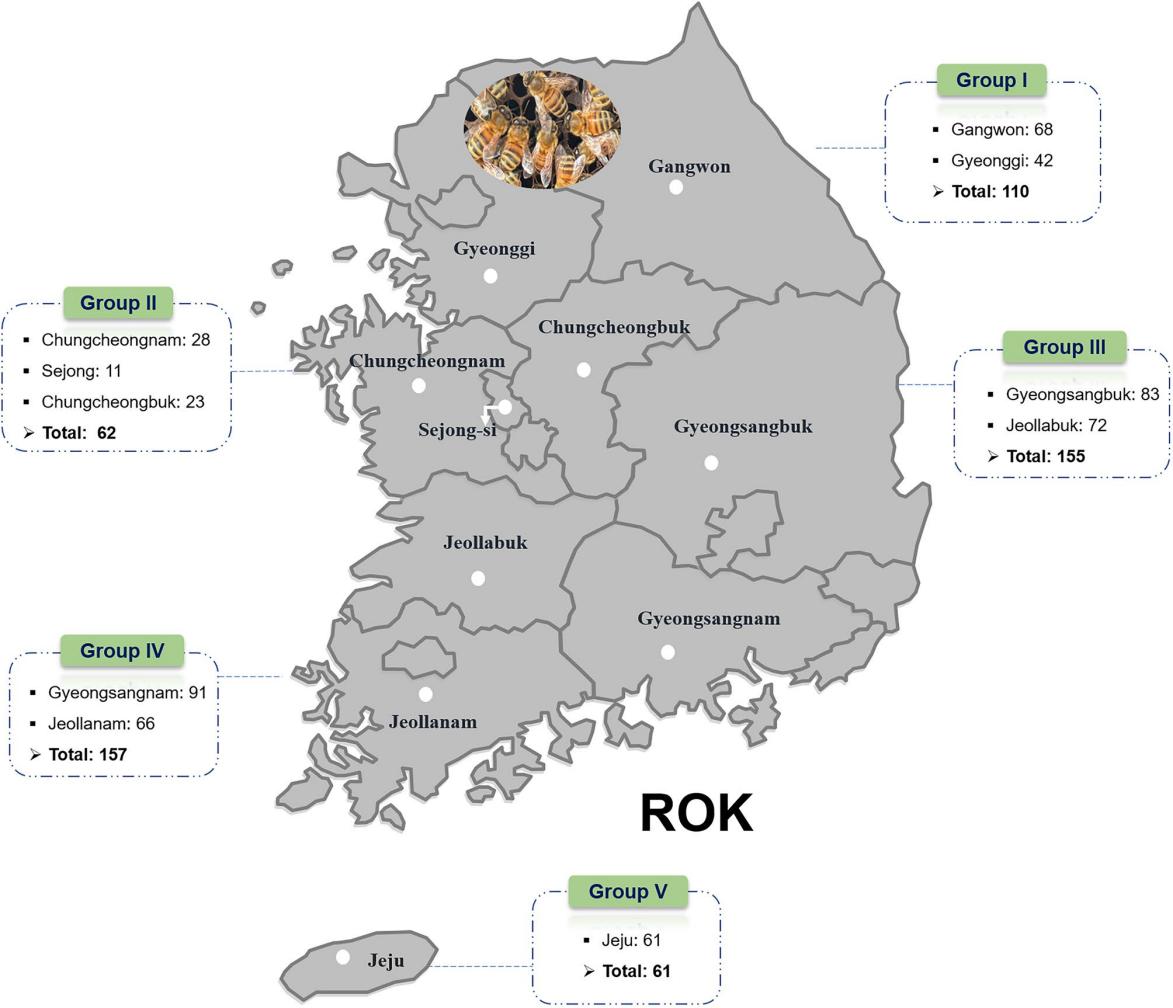
Collection of *Apis mellifera* colonies in the Republic of Korea. Honeybee samples (*n* = 545) were collected from five geographical groups within the ROK during 2022 and 2023. Group I encompassed Gangwon and Gyeonggi provinces; Group II consisted of Sejong City, Chungcheongnam, and Chungcheongbuk provinces; Group III comprised Gyeongsangbuk and Jeollabuk provinces; Group IV included Gyeongsangnam and Jeollanam provinces; and Group V pertained to Jeju province.

In this study, we aimed to investigate the presence of AmFV in honeybee colonies in the Republic of Korea (ROK) and determine its prevalence in different regions of the country (Fig. 1). Using specific primer sets targeting the baculovirus repeat open reading frame (ORF) (*Bro–*N) gene segments, we conducted PCR amplification and sequence analysis to confirm the presence of AmFV.

## Results

### Prevalence of AmFV in honeybee in ROK

**Figure 2:**
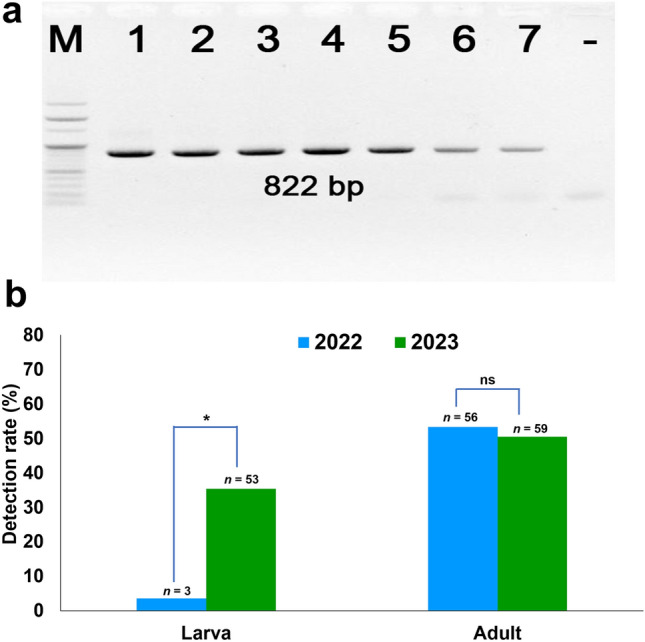
Detection of *Apis mellifera* filamentous virus (AmFV) in honeybee colonies. (**a**) PCR products of baculovirus repeat open reading frame *Bro*–N gene of AmFV in honeybee samples; M: 100-bp DNA ladder (Enzynomics, Daejeon, ROK); lanes 1 to 7: PCR performed with DNA template of honeybee samples; (−): PCR without DNA template. (**b**) *AmFV* was investigated in larvae and adult honeybees in colonies from winter, and was compared between 2022 (*n* = 189 colonies) and 2023 (*n* = 267 colonies). The total number of larvae (*n* = 84 colonies) and adult bees (*n* = 105 colonies) were collected in 2022; the total number of larvae (*n* = 150 colonies) and adult bees (*n* = 117 colonies) were collected in 2023. Statistical analysis using the likelihood ratio chi-square test was performed (*p < 0.05; “ns”, no significant difference). AmFV, *Apis mellifera* filamentous virus; PCR, polymerase chain reaction; ROK, Republic of Korea.

Low temperature and high humidity are the primary factors contributing to the emergence of various diseases in honeybee colonies^24–26^. This study investigated the prevalence of AmFV in honeybee colonies during the winters of 2022 and 2023. To achieve specific detection of AmFV, we designed a unique primer pair targeting the *Bro*–N gene segment within the viral genome. The *Bro*–N gene encodes the nucleoprotein (N), a vital protein highly conserved across AmFV strains and essential for viral replication. Figure 2a shows the PCR results for the *Bro*–N gene segment of AmFV in seven honeybee samples (Fig. 2a). Lanes 1 to 7 represent the amplified products. A band around 822 bp is present in these lanes, confirming the successful amplification of the targeted AmFV gene segment. The absence of a band in the negative control lane demonstrates the specificity of the primers which only amplify the intended AmFV *Bro*–N gene and no other DNA sequences in the samples. While not shown here, positive controls were included in a separate real-time PCR experiment to assess the overall prevalence of AmFV in the Korean honeybee population. To further verify the identity of the amplified fragment, the *Bro*–N gene segment was sequenced, and the sequences were deposited in NCBI GenBank with accession numbers OR371980.1 and OR371981.1.

Honeybee colonies were investigated for AmFV infection during winter in both 2022 and 2023. Larva showed a significant increase in infection rates between the two years (Fig. 2b). In 2022, the infection rate was 3.57% (3 out of 84 larvae). This rate significantly increased to 35.33% (53 out of 150 larvae) in 2023 (χ^2^ = 29.84, *df* = 1, *p* < 0.001). Conversely, the infection rate in adult bees remained statistically similar across the two years (*p* = 0.665). Adult bee infection rates were 53.33% (56 out of 105) in 2022 and 50.43% (59 out of 117) in 2023 (Fig. 2b). Considering all collected samples (larvae and adults combined), the overall AmFV infection rate in winter 2022 was 31.22% (59 out of 189 samples). This rate increased to 41.94% (112 out of 267 samples) in winter 2023.

**Figure 3:**
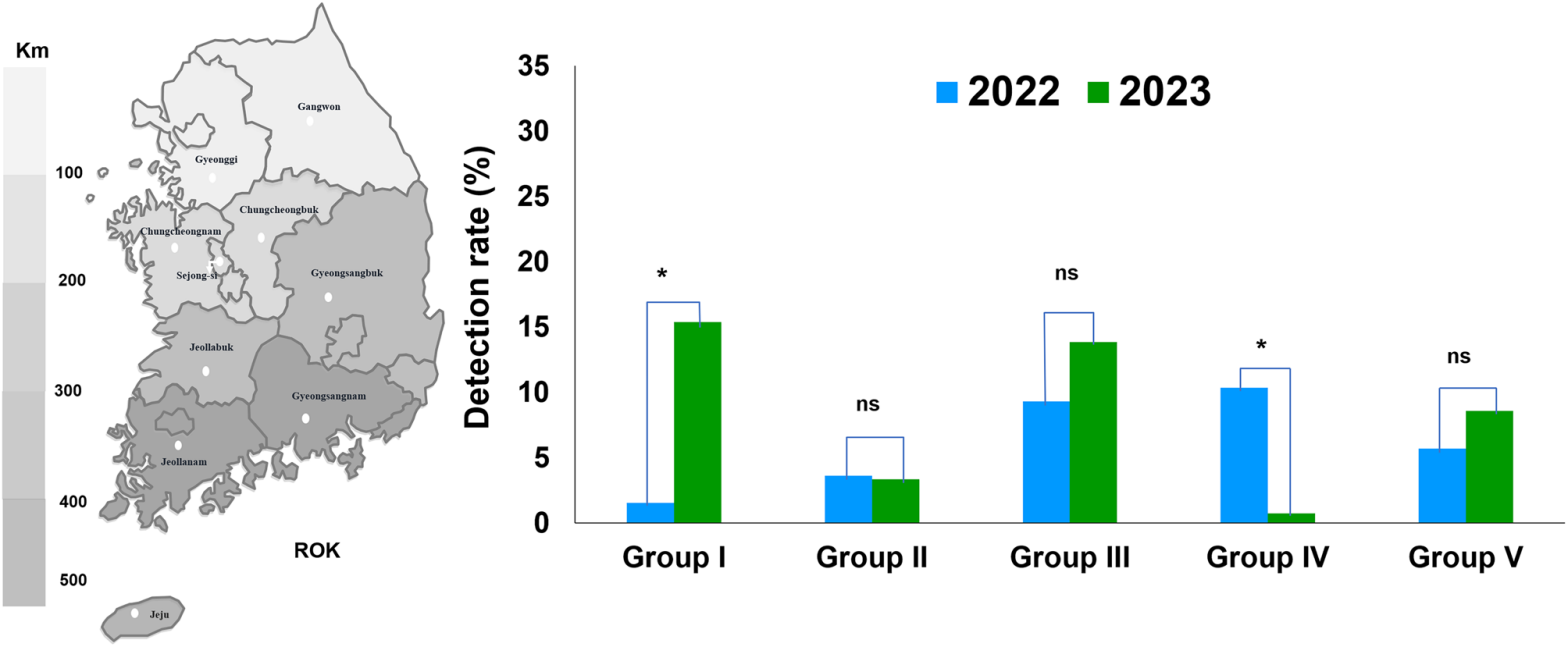
Comparison among latitude detection of *Apis mellifera* filamentous virus (AmFV). The prevalence of AmFV in Korean honeybee colonies across different latitudes during 2022 and 2023. The Korean map was divided into regions based on latitude, with color gradients from white-darker 5% to white-darker 50%. *p < 0.05, significant differences were noted with the likelihood ratio chi-square test statistical analysis; “ns”, indicates not significant.

Honeybee colonies from beekeepers in different provinces of the ROK in 2022 and 2023 were divided into five groups: Group I consisted of honeybee colonies collected from the Gyeonggi and Gangwon provinces; Group II included honeybee colonies collected from Sejong, Chungcheongbuk, and Chungcheongnam; Group III comprised honeybee colonies from Gyeongsangbuk and Jeollabuk; Group IV included honeybee colonies from Gyeongsangnam and Jeollanam; and Group V included honeybee colonies from Jeju (Fig. 1). We detected the presence of AmFV in honeybee colonies across the different provinces throughout the country. AmFV infection rates varied among groups and across years. The survey results show that the infection rate of AmFV increased significantly, by 9.88-fold, in Group I, which represented a region with lower temperatures than those in the other groups, from 1.55% in 2022 to 15.36% in 2023 *(*χ^2^ = 24.21, *df* = 1, *p* < 0.005) (Fig. 3). In Group II, there was no change in the AmFV infection rate in 2022 (3.63%) and 2023 (3.37%). In Groups III and V, the AmFV infection rate was higher increased from 9.33% (2022) to 13.86% (2023), and 5.70% (2022) to 8.61% (2023), respectively. However, the difference in infection rate of AmFV in Groups III and V were not statistically significant (*p* = 0.215 and *p* = 0.360). Notably, in Group IV, the prevalence of AmFV was significantly decreased from 10.36% in 2022 to 0.74% in 2023 (χ^2^ = 10.10, *df* = 1, *p* < 0.005). The 14-fold reduction in AmFV infection within one year may be attributed to improved beekeeping techniques implemented by beekeepers to minimize diseases in honeybee colonies in this region.

### Phylogenetic analysis of AmFV

**Figure 4:**
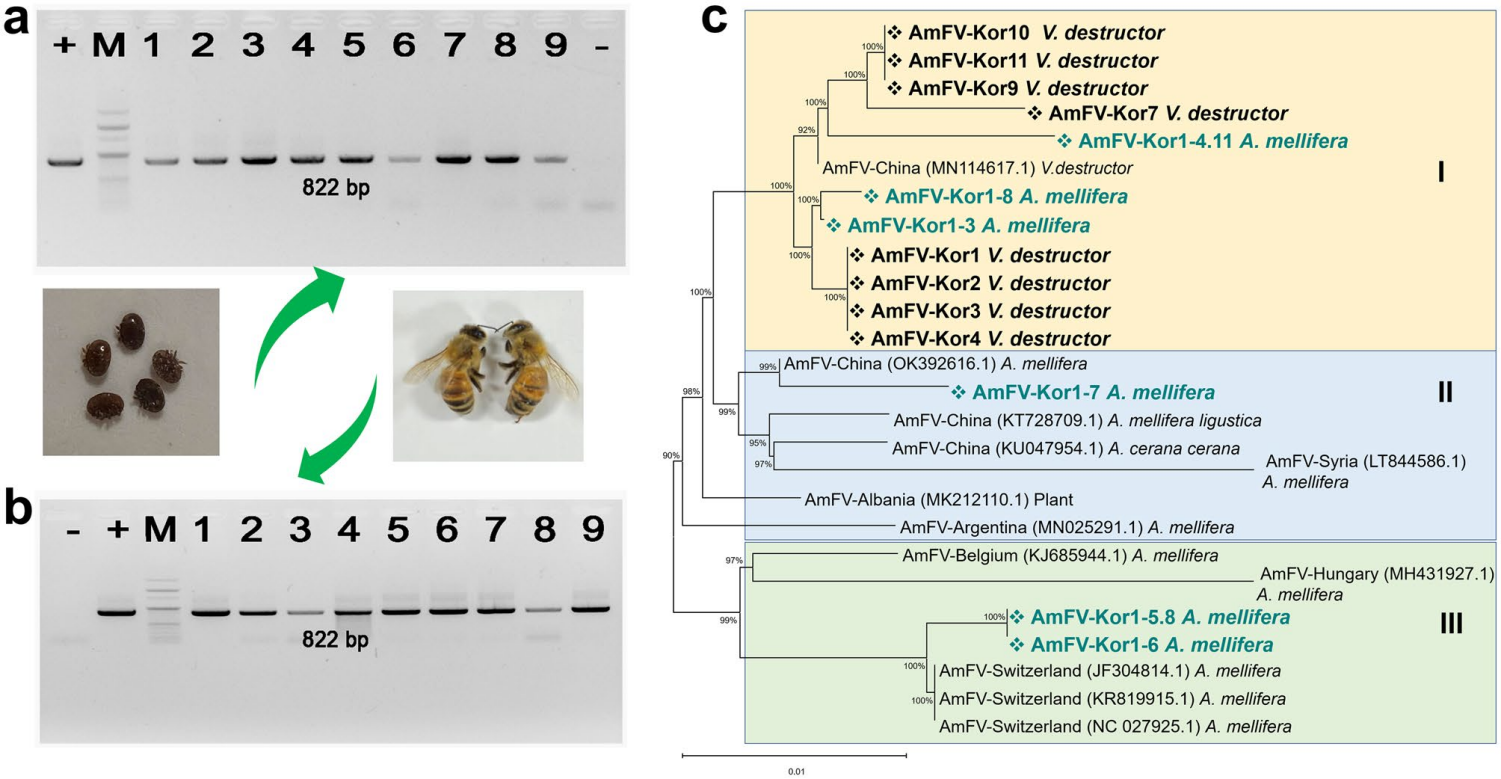
The *Bro–*N sequences of *Apis mellifera* filamentous virus isolated from honeybee and *Varroa destructor* mites. (**a**) Lanes 1–9 indicated polymerase chain reaction products obtained from *V. destructor* mite samples. (**b**) Lanes 1–9 indicated polymerase chain reaction products obtained from honeybee samples; M, 100 bp DNA ladder (Enzynomics, Daejeon, ROK); (+), positive control: PCR using plasmid of *Bro–*N gene cloned pGem*–*T vector as DNA template; (−), negative control: PCR without DNA template. (**c**) Phylogenetic tree constructed using the neighbour-joining method using *Bro–*N gene sequences (512 bp) of AmFV detected in honeybees from the ROK and from sequences from isolates obtained in other countries that had been deposited in GenBank. ROK, Republic of Korea.

A phylogenetic tree was constructed using the neighbour-joining method based on *Bro–*N gene sequences of AmFVs detected in South Korea and other countries, as reported in the GenBank database (Fig. 4). The sequences used in the analysis included isolates from various sources, including plants, *V. destructor* mites, *A. mellifera*, *A. mellifera ligustica*, and *A. cerana cerana*. The classification tree was divided into three distinct branches. Branch I consisted of AmFV isolated from *V. destructor* mites and three other strains (AmFV*–*Kor1*–*4.11, AmFV*–*Kor1*–*3, and AmFV*–*Kor1*–*8) from honeybee samples collected in Gangwon and Gyeonggi provinces of the ROK, belonging to Group I (Fig. 4a–c). These strains showed close relationships with AmFV isolates obtained from *V. destructor* mites in China. Branch II included AmFV*–*Kor1*–*7, which were isolated from honeybee samples collected in Jeollabuk province, belonging to Group III. This strain also showed close relationships with AmFV isolates from *A. mellifera, A. mellifera ligustica,* and *A. cerana cerana* in China and Syria. Branch III included samples belonging to Group IV: AmFV*–*Kor1*–*5.8, and AmFV*–*Kor1*–*6, which were isolated from honeybee samples collected in Gyeongsangnam province. These strains were closely related to AmFV isolates from *A. mellifera* in Switzerland. Phylogenetic analysis thus revealed many variants of AmFV isolated from different regions and host species in the ROK (Fig. 4c).

### Detection of AmFV in *Varroa destructor* mite

To explore the potential presence of AmFV in *V. destructor* mites, we screened TNA extracted from pools of five mites each, collected from nine AmFV-positive apiaries. The amplified *Bro*–N segment of AmFV was isolated from nine mites and honeybee colonies, as shown in Fig. 4a–b, respectively. Interestingly, AmFV was detected in all 29 mites analyzed. This finding suggests a high prevalence of AmFV within the mite population of the examined apiaries. Phylogenetic tree analysis was performed using the sequence of the *Bro–*N gene segment of AmFV, revealing that AmFV isolated from *V. destructor* mites was closely related to the corresponding honeybee species (Fig. 4c).

### Co-infection of AmFV with other pathogens

**Figure 5:**
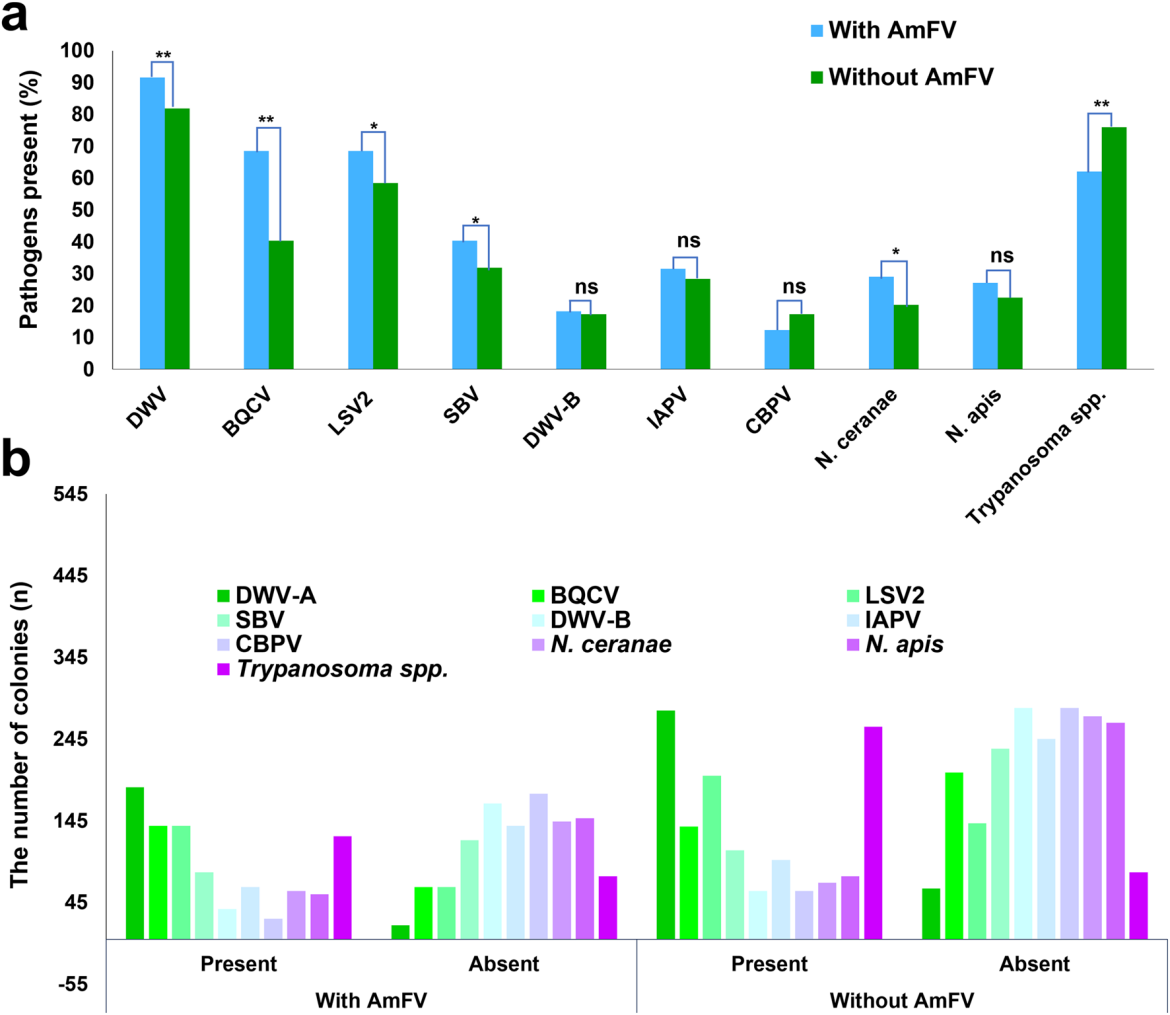
Co-occurrence of honeybee pathogens with AmFV infection. (**a**) depicted the infection rate of various pathogens in AmFV-positive and AmFV-negative colonies; (**b**) visualized the number of different pathogens detected (present or absent) within each group. Statistical analysis using the likelihood ratio chi-square test was performed. Asterisks denoted statistically significant differences (*p < 0.05, **p < 0.001), while: “ns” indicated no significant difference.

In honeybee colonies infected with AmFV, the prevalence of *N. apis* and *N. cerenae* were observed increase, as reported by Baily et al.^10^ and a higher positivety rate for BQCV during the autumn season, according to Hardmann et al.^7^. In this study, we investigated the correlation between AmFV and several co-infecting pathogens (Fig. 5a–b, Supplementary Table S1). The co-infection rate of AmFV was assessed alongside various pathogens including DWV-A, SBV, BQCV, Israeli acute paralysis virus (IAPV), Lake sinai virus 2 (LSV2), *N. ceranae, N. apis,* DWV-B (*Varroa destructor* virus 1), chronic bee paralysis virus (CBPV), and *Trypanosoma* spp. Examination of 545 colonies from 204 apiaries revealed that the prevalence of IAPV, BQCV, SBV, DWV-A, LSV2, and *N. ceranae* was higher in the AmFV*-*positive colonies (Fig. 5a–b). Specifically, the BQCV infection rate was 1.81–fold higher in colonies infected with AmFV than in colonies without AmFV. No significant correlations were observed between AmFV and three pathogens (*N. apis,* DWV-B, and IAPV). *Trypanosoma* spp. infection rate in AmFV-positive was significantly lower in AmFV-negative, showing that the AmFV-positive colonies may decrease *Trypanosoma* spp. in honeybee colonies. Also, the infection rate of CBPV was lower than in AmFV-positive but not significantly different (Fig. 5a). This observation suggests a complex interplay between AmFV and co-infecting pathogens in honeybee colonies.

## Discussion

This study provided crucial insights into the detection, phylogenetic analysis, prevalence, and co-occurrence of AmFV in honeybee colonies in South Korea. The application of molecular techniques, specifically PCR and real-time PCR, was instrumental not only in detecting AmFV in honeybee colonies but also in unravelling its international genetic relations. Determining the presence of AmFV in *V. destructor* mites collected from honeybee colonies positive for AmFV demonstrates the widespread distribution of AmFV. Additionally, by the investigation of bee larvae, adult bees, and *Varroa* mites, this research provides new information and disease status for beekeepers in ROK.

The information of a distinct branch in the phylogenetic tree based on the *Bro–*N gene segment of AmFV isolated from Korean honeybee colonies and from *Varroa* mites in China suggests a close relationship between the viruses in these two regions. Furthermore, we observed that certain AmFV strains isolated from honeybee colonies in the ROK exhibited genetic affinities with strains from Switzerland. The diversity of AmFV strains has previously been reported for various regions of China and Argentina^17,27^. The results indicate a high degree of genetic diversity among AmFV isolates, with many variants identified within honeybee apiaries across ROK. Our analysis of the *Bro–*N gene and a hypothetical protein coding sequence of AmFV (Supplementary Fig. S1) revealed nucleotide variations, suggesting the presence of different AmFV strains among the samples. This finding suggests a potentially long-established presence of AmFV in the Korean bee population, warranting further investigation into the origin and potential impact of the AmFV variants. Further in-depth investigations are necessary to determine the fundamental causes of pathogen emergence through the analysis of the genetic phylogeny of the strains.

We also investigated the prevalence of AmFV in honeybee colonies in South Korea. Analysis of the samples collected in 2022 and 2023 revealed a relatively high prevalence of AmFV. Our study detected AmFV in both larval and adult honeybees during winter surveys (Fig. 2b). This aligns with previous findings by Gauthier et al.^11^ who reported AmFV presence in various honeybee life stages, including queen ovaries, eggs, sperm, pupae, and worker bees. However, our study revealed a significant difference in infection rates between larvae across the winter seasons of 2022 and 2023 (χ^2^ = 324.24, *df* = 1, *p* < 0.001). It may explain this observed difference in two ways. First, discrepancies in the sampling process could play a role. Second, the viral load in larvae collected in 2022 might be not enough to be detected, pushing it to decrease the detection limit of our method. This highlights the importance of meticulous sampling techniques when analyzing honeybee pathogens. Future studies should investigate AmFV levels across different developmental stages of honeybees, along with the exploration of potential transmission routes within the hive. As climate can influence the infection rates of viral diseases, we analyzed the infection rate according to latitude. The comparison of AmFV infection rates among the five different groups in the ROK based on variations in weather conditions revealed no significant differences in infection rates among the surveyed regions (*p* > 0.05). However, genetic diversity across the studied regions was notable, with distinct genotypic clusters observed in the pathogen population. This finding suggests potential geographic isolation or adaptation to local environmental factors, warranting further investigation into the specific genetic variations and their potential functional significance.

In China, the prevalence of AmFV in beehives ranges from 10 to 80%^8,20^. Similarly, a study conducted in Argentina, representing eight provinces within the country, reported the presence of AmFV in 61% of cases^17^. Our results indicated a relatively high prevalence (approximately 41.94% in 2023) and widespread distribution of AmFV in Korean honeybee colonies. The detection of AmFV variants in both larvae and adult bees indicates its potential to establish persistent infections within honeybee colonies, potentially leading to higher infection rates. The observed differences in AmFV prevalence across regions require further investigation. Potential contributing factors include environmental conditions, beekeeping practices, and interactions with other pathogens. Since temperature variations within the groups were not fully determined, future studies conducted throughout different seasons are crucial to elucidate the relationship between climatic fluctuations and AmFV infection levels in honeybee colonies.

Recent studies have suggested that mites such as *V. destructor* and *Tropilaelaps mercedesae* play significant roles in the transmission of viral diseases like DWV and IAPV among honeybees^28–31^. *V. destructor* and *Tropilaelaps mercedesae* mites are two disease-transmitting vectors among honeybees and have been reported to have a high infection rate in Korean honeybee apiaries^32–34^. *Tyrophagus curvipenis* and *Neocypholaelap* spp. mites have also been reported to detect honeybee pathogens in these recently identified mites in Korean apiaries^35,36^. AmFV hasn’t been detected in *T. curvipenis*^35^ and *Neocypholaelap* spp.^36^ in the previous studies. Meanwhile, our findings and previous reports^11,37,38^ confirm the presence of AmFV in *Varroa* mites (Fig. 4a). Furthermore, the high detection rate in mites could be due to two possibilities such as passive acquisition and potential vector role. As Gauthier et al.^11^ mentioned, AmFV releases a large number of particles into the bee hemolymph, and was found in queen ovaries, eggs, drone sperm, larvae, and adult bees suggesting that potential for vertical transmission of this virus. During feeding, *Varroa* mites might passively acquire these viral particles without necessarily being infected themselves. Alternatively, *Varroa* mites could act as reservoirs or vectors for AmFV transmission between bees. Our study also detected AmFV in *Varroa* mites. Unfortunately, mites could act as passive carriers or reservoirs, facilitating AmFV transmission between bees. While we cannot definitively confirm AmFV replication within mites due to the lack of full genome isolation, their association with AmFV suggests a potential role in this process. This finding is significant because *Varroa* mites are established honeybee parasites and known vectors for other bee viruses. It adds to the growing body of evidence implicating these mites in spreading viral pathogens among honeybees. Further research is needed to elucidate the specific mechanisms of AmFV transmission by mites. This emphasizes the importance of implementing effective mite control measures to limit mite populations and their potential role as viral vectors. By reducing mite infestations, we may be able to mitigate the impact of AmFV on honeybee health and colony loss.

This study revealed that AmFV infection was associated with an increased prevalence of *Nosema* spp. in honeybee colonies. Our data demonstrated a significantly higher infection rate of *N. ceranae* in AmFV*-*positive colonies than in AmFV*-*negative colonies (χ^2^ = 5.60, *df* = 1, *p* < 0.05). However, other studies reported that no relationship between the presence of AmFV and *N. ceranae* from Switzerland, the US, and Europe^7,23,39,40^. Differences in sampling methods, nucleic acid extraction procedures, and PCR protocols may affect the sensitivity and specificity of detection. Discrepancies in geographical location, beekeeping practices, and the prevalence of both AmFV and *N. ceranae* in honeybee populations may contribute to the observed differences. Although our primer sequences may target conserved regions of AmFV, there is still the possibility of mutations occurring in strains detected within the Korean honeybee population. Accurate assessment would necessitate sequencing honeybee pathogens to provide more comprehensive picture of the prevalence of AmFV and its interactions with honeybee pathogens including *Nosema* in different regions. Furthermore, although a slight increase in the infection rate of *N. apis* in AmFV-positive colonies was noted, the difference was not statistically significant (*p* = 0.228) (Fig. 5a–b). The lack of a clear association between *N. apis* and AmFV is in line with investigations in Swiss colonies^7,41^ and in contrast to previous findings from England^10^. Therefore, the relationship between AmFV and *Nosema* infection warrants further investigation. AmFV has been found to be associated with DWV-A, SBV, BQCV, and CBPV, which were identified in the spring, summer, and fall^7,11^. Hartmann et al.^7^ reported a notable correlation between the AmFV and BQCV during the fall season. Therefore, in this study, the rapid increases in SBV and BQCV by 2023 may have been associated with AmFV.

Trypanosomatidae (*Crithidia mellificae/Lotmaria passim,* and *Trypanosoma* spp.) are widely distributed pathogens in insects, including honeybees^42,43^. *C. mellificae/L. passim* has been associated with winter colony mortality^44^. In this study, we investigated the correlation between AmFV and *Trypanosoma* spp*.*; however, we found that the *Trypanosoma* ssp. infection rate was not correlated with AmFV, as the *Trypanosoma* ssp. infection rate in AmFV-positive colonies was lower than that in AmFV-negative colonies (χ^2^ = 12.00, *df* = 1, *p* < 0.05). Therefore, seasonal co-infection relatedness needs to be investigated in more detail with samples from more honeybee colonies collected in different seasons in the ROK. The primary limitation of this study stems from the different conditions and technical care methods among beekeepers have posed challenges in comparing infection rates among areas categorized by latitude. Furthermore, the prevalence of new pathogens in beekeeping in the ROK remains inadequately understood. Therefore, further study is essential to comprehend the prevalence levels, the relationship between honeybee pathogens, and the factors contributing to recent occurrences of honeybee colony losses in the ROK.

This study provided evidence for the presence of AmFV in honeybee samples and *V. destructor* mites in the ROK. The identification of AmFV in both honeybees and mites suggests a potential role for mites in the transmission and spread of the virus within honeybee colonies. AmFV infection can potentially increase the prevalence of LSV2, SBV, DWV-A, and BQCV in honeybee apiaries. These findings contribute to our understanding of AmFV distribution and emphasize the need for continued research and management efforts to protect honeybee populations from newly identified viral infections.

## Materials and methods

### Honeybee colonies sampling

This study was conducted in 2022 and 2023 for the investigation of the prevalence of AmFV in honeybee colonies across ROK. In total, 545 colonies were collected from 204 beekeeping farms in nine provinces. Many of these apiaries were located near hills or mountains, which provided abundant natural food sources for bees, and they were fixed in one place for honey collection. The health and disease status of bee colonies in nine provinces were not the same. The honeybee samples were collected with divided into five groups in ROK (Fig. 1). Every hive was carefully inspected and the comb frame was removed without disturbing the queen. Adult bee samples (*n* = 10–30) were collected in 50 mL Falcon tubes and larva samples (*n* = 30) were collected using forceps from three randomly selected locations within the hive. All samples were transported to − 80 °C freezer for further analysis at Laboratory Parasitic and Honeybee Diseases of the Animal and Plant Quarantine Agency, ROK.

### Varroa destructor mite sampling

*Varroa* mite infection levels were assessed in honey bee colonies by shaking powdered sugar with 300 bees per colony^45^. The collected mites were then transported to the same bee storage and frozen at − 80 °C for further experiments. To investigate the potential role of mites in AmFV transmission, *Varroa* mite samples were specifically selected from 29 AmFV-positive honey bee colonies to determine the presence or absence of AmFV in the mite population. This study did not require ethical approval since it did not include vertebrates or cephalopods.

### Total nucleic acid extraction

The total nucleic acid (TNA) from honeybee samples and *V. destructor* mite samples were extracted using the Maxwell^®^ RSC viral total nucleic acid purification kit (Promega, Madison, WI, USA). *V. destructor* mite samples were washed three times with 70% ethanol and distilled water prior to nucleic acid extraction. The mite (five mites/tube) samples and honeybee samples (two bees/tube) were homogenised using a Precellys 24 tissue homogeniser (Bertin Instruments, Montigny*–*le Bretonneux, France) for three 15 s cycles at 5000 rpm in 1 mL phosphate-buffered saline solution. Subsequently, 200 μL of lysis buffer mixed with 20 μL of proteinase K solution, and 200 μL of the homogenate were combined and incubated at 56 °C for 10 min. The nucleic acids were then purified using the automated Maxwell^®^ RSC instrument following the manufacturer's instructions. Finally, 60 μL of the purified total nucleic acid was acquired from each sample. Total nucleic acids extraction of the honeybee and mite samples were stored at − 20 °C until used. The cDNA and TNA concentration were quantified using Thermo Scientific Nanodrop 2000c spectrophotometer (Thermo Fisher Scientific, USA).

### Pathogen detection using molecular biology approaches

The primer pairs used for real-time PCR for detecting AmFV were designed based on the known sequences of AmFV isolated from honeybee samples^35^. Another primer pair was designed for the phylogenetic analysis of AmFV by comparison to *Bro–*N gene sequences of AmFV strains published in GenBank. The primer pairs used in this study are listed in Table 1.

**Table 1:** Primers used in this study.

Name of primers	Sequences (5′ to 3′)	Amplicon size (bp)	Note	References
AmFV1*–*For	CCGCAGGCTTCAACGAATTA	122	Used in real-time PCR for detection	This study
AmFV1*–*Rev	GTCTCGGGTAACCACGTACT			
AmFV2*–*For	CTTGAATTTGTAGAATGGA	822	Used in PCR for nucleotide sequencing	
AmFV2*–*Rev	CTAATCGAGGTGTCCGAGGT			

To investigate co-infection with AmFV, we screened honeybee samples for the presence of various pathogens using real-time PCR and real-time RT-PCR. These pathogens included SBV, DWV-A, BQCV, LSV2, DWV-B, CBQV, IAPV, *Trypanosoma* spp., *N. ceranae,* and *N. apis*. As described previously, *C*_*t*_ values ≤ 35 were considered indicative of a positive detection^26,34,35^. The primers used for the amplification of pathogens were shown in Supplementary Table S2^35,46^. Details on the specific kits utilized for each pathogen are available from iNtRON and Bio-Rad companies. These kits include LiliF ABPV/KBV/IAPV/CBPV real*-*time RT-PCR Kit, LifiF sacbrood virus (SBV)/KSBV/deformed wing virus (DWV)/BQCV real*-*time RT-PCR Kit (iNtRON Biotechnology, Inc., Seongnam, ROK), iTaq Universal SYBR Green One-Step Kit (Bio-Rad, USA), SsoAdvanced Universal SYBR Green Supermix [Bio-Rad, USA], and Pobgen bee pathogen detection kit (Postbio Co. Ltd., Namyangju, ROK).

### Polymerase chain reaction

PCR was conducted using AccuPower^®^ Taq PCR PreMix & Master Mix kit (Bioneer, ROK) with a 20 μL reaction mixture, consisting of 1 μL cDNA (~ 80 ng) or 1 μL TNA (~ 40 ng), 1 μL of each primer (5 pMol), and 17 μL of ddH_2_O. Optimal cycling conditions were as follows: an initial denaturation step at 94 °C for 2 min, followed by 30 cycles at 94 °C for 30 s, at 55 °C for 30 s, and at 72 °C for 30 s, and a final extension at 72 °C for 10 min. PCR products were visualised by electrophoresis on a 1% agarose gel.

### Real-time polymerase chain reaction

Real-time PCR was performed using SYBR^®^ Green Supermix (Bio-Rad, Hercules, CA, USA) in a reaction volume of 20 μL. The reaction mixture consisted of 10 μL of 2 × SYBR Mix, 0.5 μL of each primer, 1 μL of TNA (~ 40 ng), and 8 μL of ddH_2_O. Optimal cycling conditions were as follows: an initial denaturation step at 94 °C for 2 min, followed by 40 cycles of denaturation at 94 °C for 30 s, annealing at 55 °C for 30 s, and extension at 72 °C for 30 s. To verify the specificity of the PCR amplification, a melting-curve analysis was performed. The positive control was constructed through the amplification of *Bro*–N gene of AmFV-infected honeybees in ROK, followed by cloning into pGem–T vector and transferred to *Escherichia coli* DH5α. Negative and positive controls were included for each run. Samples with a *C*_*t*_ value of ≤ 35 and consistent melting curves were considered positive.

### Sequence analysis and data analysis

PCR products were purified and sequenced by Cosmogenetech (Daecheon, ROK). The obtained nucleotide sequences of the *Bro–*N gene were aligned with sequences in Genbank (http://www.ncbi.nlm.nih.gov/Genbank) using the Nucleotide Basic Local Alignment Search Tool (BLASTn). For phylogenetic analysis, sequences were aligned using ClustalW in BioEdit9 software^47^. Phylogenetic analysis was performed using the neighbour-joining method with 1000 bootstrap replicates using MEGA 11 software^48^. The analysis was based on a 790-bp fragment of the *Bro–*N gene and included sequences from AmFV isolates reported from various countries, such as Switzerland (NCBI Accession No: JF304814.1, KR819915.1, and NC_027925.1), Belgium (KJ685944.1), China (NCBI Accession No: OK392616.1, KU047954.1, KT728709.1, and MN114617.1), Hungary (NCBI Accession No: MH431927.1), Argentina (NCBI Accession No: MN025291.1), Albania (NCBI Accession No: MK212110.1), and Syria (NCBI Accession No: LT844586.1).

The likelihood ratio chi-square test of contingency was used to compute the probability of equal pathogens in two independent groups, and one-way analysis of variance (ANOVA) was used to compare more than two group. Statistical significance was set at *p* < 0.05.

### Supplementary Information

Supplementary Information.

Supplementary Figure S2.

Supplementary Figure S3.

## Data Availability

All relevant data are available within the paper. The sequencing data of baculovirus repeat open reading frame*–*N gene in this study were deposited in the NCBI under the OR371981.1, OR371980.1, and OR371979.1 accession numbers.
