# Supplementary material for: First detection and prevalence of Apis mellifera filamentous virus in Apis mellifera and Varroa destructor in the Republic of Korea
Source: Sci Rep. 2024 Jun 19;14:14105. doi: 10.1038/s41598-024-64882-z (PMC11189470; doi:10.1038/s41598-024-64882-z)
Supplement: Supplementary file 1 — Supplementary Information. [file 41598_2024_64882_MOESM1_ESM.doc]

*Supplementary Information for*

**First Detection and Prevalence of *Apis mellifera* Filamentous Virus in *Apis mellifera* and *Varroa destructor* in the Republic of Korea**

Thi-Thu Nguyen, Mi-Sun Yoo, Hyang-Sim Lee, A-Tai Truong, So-Youn Youn, Se-Ji Lee, Jaemyung Kim & Yun Sang Cho

**Table S1.** Pathogen detection in the AmFV-positive and AmFV-negative honeybee colonies

| **Pathogen** | **With AmFV** | | | **Without AmFV** | | | ***P*-value** |
| --- | --- | --- | --- | --- | --- | --- | --- |
|  | **Present** | **Absent** | **Proportion present (%)** | **Present** | **Absent** | **Proportion present (%)** |  |
| **DWV-A** | 186 | 17 | 91.63 | 280 | 62 | 81.87 | 0.002 |
| **BQCV** | 139 | 64 | 68.47 | 138 | 204 | 40.35 | < 0.001 |
| **LSV2** | 139 | 64 | 68.47 | 200 | 142 | 58.48 | 0.020 |
| ***Trypanosoma* spp.** | 126 | 77 | 62.07 | 260 | 82 | 76.02 | 0.001 |
| **SBV** | 82 | 121 | 40.39 | 109 | 233 | 31.87 | 0.044 |
| ***N. ceranae*** | 59 | 144 | 29.06 | 69 | 273 | 20.18 | 0.018 |
| ***N. apis*** | 55 | 148 | 27.09 | 77 | 265 | 22.51 | 0.228 |
| **DWV-B** | 37 | 166 | 18.23 | 59 | 283 | 17.25 | 0.773 |
| **CBPV** | 25 | 178 | 12.32 | 59 | 283 | 17.25 | 0.123 |
| IAPV | | 64 | 139 | 31.53 | 97 | 245 | 28.36 |  | | --- | --- | --- | --- | --- | --- | --- | | 139 | 31.53 | 97 | 245 | 28.36 | 0.434 |

Values represent the number of honeybee colonies (*n* = 203 AmFV-positive colonies, *n* = 342 AmFV-negative colonies). Data were recorded for 545 colonies in 204 apiaries. SBV, sacbrood virus; BQCV, black queen cell virus; CBPV, chronic bee paralysis virus; DWV-A, deformed wing virus; DWV-B, deformed wing virus B (*Varroa destructor* virus); LSV2, Lake Sinai virus 2; *Nosema ceranae (N. ceranae)*; *Nosema apis (N. apis)*. The likelihood ratio chi-square test of contingency was used to compute the probability of equal pathogen incidence in AmFV-positive and AmFV*-*negative colonies.

**Table S2. Primers and probes used for the d**etection of pathogens

| **No.** | **Target** | **Primer** | **Sequence (5ꞌ→3ꞌ)** | **Amplicon size (bp)** | **Ref.** |
| --- | --- | --- | --- | --- | --- |
| **1** | SBV | SBV-F | AGAAGACATTTGATACAGTGGACTC | 131 | 1 |
|  |  | SBV-R | GGAATTCCAGATTCTTCGTCCAC |  |  |
|  |  | Probe | FAM–GATTTGTTTAATGGTTGGGTTTCTGGTA–BHQ-1 |  |  |
| **2** | DWV-A | DWV-F | TTCAACTCGGCTTTCTACGG | 170 |  |
|  |  | DWV-R | GTGTCTTTTTCTCTTTCTGACACC |  |  |
|  |  | Probe | ROX- ATGTCAACATTGGTATGCTCCGTTGAC- BHQ-2 |  |  |
| **3** | BQCV | BQCV-F | CCTTTGGCAATAGAACAAATACC | 143 |  |
|  |  | BQCV-R | GTGGCTATATCGAGATTATTCCG |  |  |
|  |  | Probe | Cy5- AGTCGCAGAGTTCCAAATACCGTACTATG- BHQ-3 |  |  |
| **4** | CBPV | CBPV-F | CGCAAGTACGCCTTGATAAAGAAC | 101 |  |
|  |  | CBPV-R | ACTACTAGAAACTCGTCGCTTCG |  |  |
|  |  | Probe | Cy5- TCAAGAACGAGACCACCGCCAGTTC- BHQ-3 |  |  |
| **5** | IAPV | IAPV -F | TGCCCTATTTAGGGTGAGGAG | 245 |  |
|  |  | IAPV -R | GGAGTTTCCACATCATGAAAGG |  |  |
|  |  | Probe | ROX-ACTAGTGAGAACTCGGTTGAGACCCAAG-BHQ-2 |  |  |
| **6** | AmFV | AmF-For | CACGAAAGCCCTCAAATCGT | 155 | 2 |
|  |  | AmF-Rev | TGTAATTCAAAATTTTTTCG |  |  |
| **7** | LSV2 | LSV2-F | GCGGGTAAATCCACTAAGGC | 218 |  |
|  |  | LSV2-R | ATTACGCACCTTCGTTTGGG |  |  |
| **8** | DWV-B | VDV-1 F | GCCCTGTTCAAGAACATG | 413 | 3 |
|  |  | VDV-1 R | CTTTTCTAATTCAACTTCACC |  |  |
| **9** | *Nosema ceranae* | *N.ceranae*-F | CAATATTTTATTATTTTGAGAGA | 223 | This study |
|  |  | *N.ceranae*-R | TATATCTATTGTATTGCGCGTGCA |  |  |
| **10** | *Nosema apis* | *N. apis*-F | CAATATTTTATTGTTCTGCGAGG | 241 |  |
|  |  | *N. apis*-R | AAAGTCTATTGTATTGCGCGTGCT |  |  |
| **11** | *Typanosoma* spp. | Try-For | GGGCGGCATATCTGTTACAC | 276 | 2 |
|  |  | Try-Rev | AAGAGCCGACATCGAAGGAT |  |  |

Abbreviations: SBV: Sacbrood virus; BQCV: black queen cell virus; CBPV: chronic bee paralysis virus; DWV-A: deformed wing virus; DWV-B: *Varroa destructor* virus 1, VDV-1); AmFV: *Apis mellifera* filamentous virus.


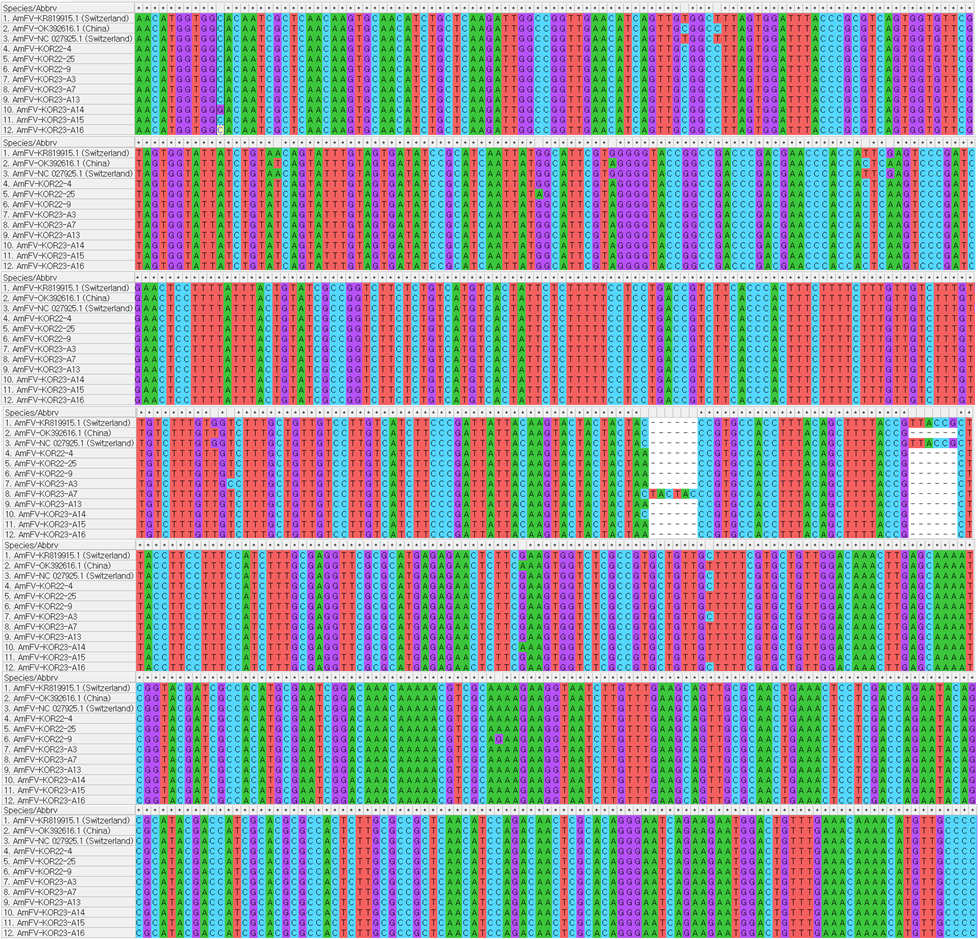


**Figure S1. Multiple alignments of the 714 bp hypothetical protein coding sequence of** ***Apis mellifera* filamentous virus**. A comparison was conducted between the hypothetical protein coding sequence of AmFV isolated from the ROK in this study and the published sequences in GenBank (NCBI accession nos.: KR819915.1 and NC_027925.1 from Switzerland; OK392616.1 from China).

**References**

1 Truong, A. T. *et al*. Prevalence of honey bee pathogens and parasites in South Korea: A five-year surveillance study from 2017 to 2021. *Heliyon* **9**, e13494 (2003).

2 Nguyen, T. T. *et al*. First identification of *Tyrophagus curvipenis* (Acari: Acaridae) and pathogen detection in *Apis mellifera* colonies in the Republic of Korea. *Sci. Rep.* **13**, 9469 (2023).

3 Radzevičiūtė, R. *et al*. Replication of honey bee-associated RNA viruses across multiple bee species in apple orchards of Georgia, Germany and Kyrgyzstan. *J. Invertebr. Pathol.* **146**, 14–23 (2017).
