# Supplementary material for: First detection and prevalence of Apis mellifera filamentous virus in Apis mellifera and Varroa destructor in the Republic of Korea
Source: Sci Rep. 2024 Jun 19;14:14105. doi: 10.1038/s41598-024-64882-z (PMC11189470; doi:10.1038/s41598-024-64882-z)
Supplement: Supplementary file 2 — Supplementary Figure S2. [file 41598_2024_64882_MOESM2_ESM.pdf]

Supplementary Information 1

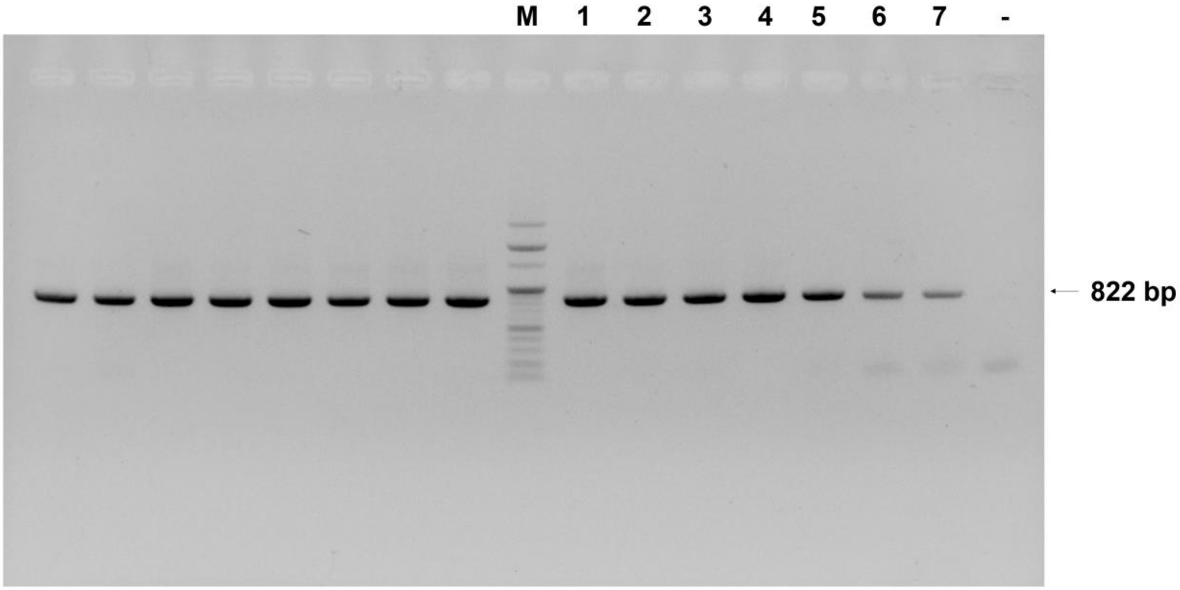

Supplementary Figure 2. **Original gel used to prepare Figure 2a.** M: 100-bp DNA ladder (Enzynomics, Daejeon, ROK); lanes 1 to 7: PCR performed with DNA template of honeybee samples; (−): PCR without DNA template. AmFV, *Apis mellifera* filamentous virus; PCR, polymerase chain reaction; ROK, Republic of Korea.
