# Supplementary material for: First detection and prevalence of Apis mellifera filamentous virus in Apis mellifera and Varroa destructor in the Republic of Korea
Source: Sci Rep. 2024 Jun 19;14:14105. doi: 10.1038/s41598-024-64882-z (PMC11189470; doi:10.1038/s41598-024-64882-z)
Supplement: Supplementary file 3 — Supplementary Figure S3. [file 41598_2024_64882_MOESM3_ESM.pdf]

Supplementary Information 2

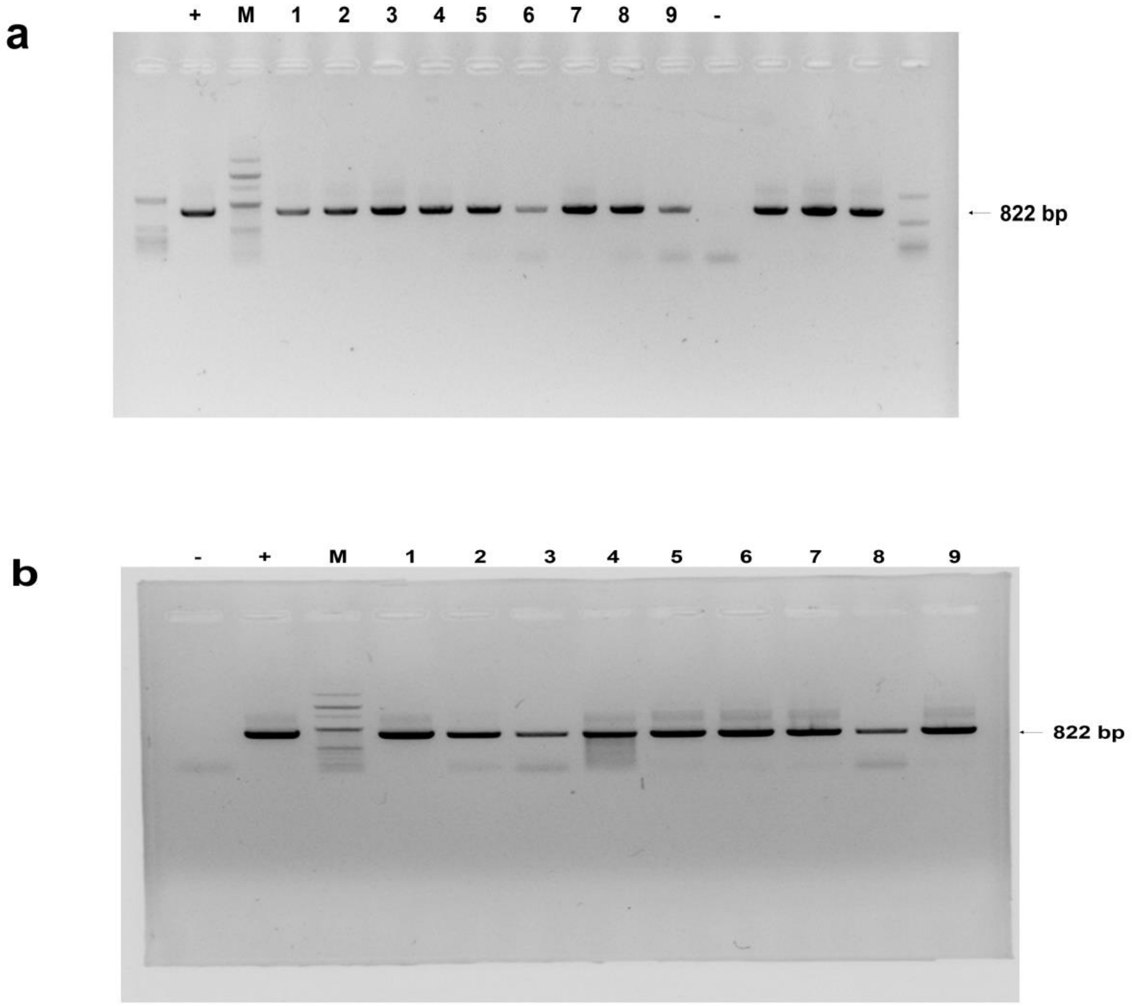

Supplementary Figure S3. **Original gels used to prepare Figure 4a–b.** *Apis mellifera* filamentous virus (AmFV) detection in *Varroa destructor* mites and honeybee samples. (**a**) Lanes 1–9 indicate polymerase chain reaction products obtained from *V. destructor* mite samples, (**b**) lanes 1–9 indicate polymerase chain reaction products obtained from honeybee samples; M, 100-bp DNA ladder (Enzynomics, Daejeon, ROK); (+), positive control: PCR using plasmid of *Bro–N* gene cloned pGem–T vector as DNA template; (−), negative control: PCR without DNA template.
